# C1q and HBHA-specific IL-13 levels as surrogate plasma biomarkers for monitoring tuberculosis treatment efficacy: a cross-sectional cohort study in Paraguay

**DOI:** 10.3389/fimmu.2024.1308015

**Published:** 2024-03-13

**Authors:** Graciela Russomando, Diana Sanabria, Chyntia Carolina Díaz Acosta, Leticia Rojas, Laura Franco, Rossana Arenas, Giovanni Delogu, Mame Diarra Bousso Ndiaye, Rim Bayaa, Niaina Rakotosamimanana, Delia Goletti, Jonathan Hoffmann

**Affiliations:** ^1^ Instituto de Investigaciones en Ciencias de la Salud, National University of Asunción, Asunción, Paraguay; ^2^ Hospital General de San Lorenzo, Ministerio de Salud Pública y Bienestar Social (MSPyBS), Asunción, Paraguay; ^3^ Dipartimento di Scienze Biotecnologiche di Base, Cliniche Intensivologiche e Perioperatorie – Sezione di Microbiologia, Università Cattolica del Sacro Cuore, Rome, Italy; ^4^ Unité des Mycobactéries, Institut Pasteur de Madagascar, Antananarivo, Madagascar; ^5^ Medical and Scientific Department, Fondation Mérieux, Lyon, France; ^6^ Translational Research Unit, Department of Epidemiology and Preclinical Research, “L. Spallanzani” National Institute for Infectious Diseases (INMI), IRCCS, Rome, Italy

**Keywords:** pulmonary tuberculosis, host biomarkers, treatment immunomonitoring, plasma protein signature, immunodiagnostic

## Abstract

**Introduction:**

New diagnostic tools are needed to rapidly assess the efficacy of pulmonary tuberculosis (PTB) treatment. The aim of this study was to evaluate several immune biomarkers in an observational and cross-sectional cohort study conducted in Paraguay.

**Methods:**

Thirty-two patients with clinically and microbiologically confirmed PTB were evaluated before starting treatment (T0), after 2 months of treatment (T1) and at the end of treatment (T2). At each timepoint plasma levels of IFN-y, 17 pro- and anti-inflammatory cytokines/chemokines and complement factors C1q, C3 and C4 were assessed in unstimulated and Mtb-specific stimulated whole blood samples using QuantiFERON-TB gold plus and recombinant *Mycobacterium smegmatis* heparin binding hemagglutinin (rmsHBHA) as stimulation antigen. Complete blood counts and liver enzyme assays were also evaluated and correlated with biomarker levels in plasma.

**Results:**

In unstimulated plasma, C1q (P<0.001), C4 (P<0.001), hemoglobin (P<0.001), lymphocyte proportion (P<0.001) and absolute white blood cell count (P=0.01) were significantly higher in PTB patients at baseline than in cured patients. C1q and C4 levels were found to be related to *Mycobacterium tuberculosis* load in sputum. Finally, a combinatorial analysis identified a plasma host signature comprising the detection of C1q and IL-13 levels in response to rmsHBHA as a tool differentiating PTB patients from cured TB profiles, with an AUC of 0.92 (sensitivity 94% and specificity 79%).

**Conclusion:**

This observational study provides new insights on host immune responses throughout anti-TB treatment and emphasizes the role of host C1q and HBHA-specific IL-13 response as surrogate plasma biomarkers for monitoring TB treatment efficacy.

## 1 Introduction

Tuberculosis (TB), an infectious disease caused by *Mycobacterium tuberculosis* (Mtb), remains a major global health problem with around 10.6 million cases in 2022 (1).

Pulmonary tuberculosis (PTB) occurs in around 80% of TB cases and can be treated with WHO-approved treatment regimens, based on the resistance profile of Mtb to first- and second-line drugs (2, 3). TB treatment has the potential to cure the patient, restore quality of life and productivity, while preventing death, relapse, and transmission of drug-resistant strains. Completing long-term treatment represents a major challenge for patients and clinicians alike, particularly for patients undergoing treatment for multidrug-resistant tuberculosis (MDR-TB), for whom treatment is often associated with side effects. Treatment monitoring tools are needed to determine, from the initial phase of treatment, whether the patient is responding correctly to treatment.

TB treatment monitoring is currently assessed by clinical evaluation of the patients, as well as by analysis of sputum samples for microbiological testing at the second month and at the end of treatment (2). However, sputum samples often contain an insufficient number of bacteria, which has an impact on the performance of sputum smear microscopy and the molecular tests recommended by the WHO for the rapid diagnosis of tuberculosis (mWRD) (4). Sputum culture remains the gold-standard diagnostic test, but it takes time and results are only available after 3 to 6 weeks.

Non-sputum-based assays, such as blood tests, are considered a potential valid alternative or substitute approach for active TB diagnosis, triage and treatment monitoring (5–7). Among existing blood tests for TB, interferon-γ release assays (IGRA) remain specifically useful for the identification of latent tuberculosis infection and (8–10) and their usefulness as a treatment monitoring tool has been also evaluated (11–13). Experimental test based on the response to recombinant *Mycobacterium smegmatis* heparin binding hemagglutinin(rmsHBHA) have been shown to discriminate between TB disease and infection in HIV-uninfected and infected individuals (11, 12, 14–16). Major advances in transcriptomics, metabolomics and proteomics have made it possible to develop tools for monitoring the efficacy of TB treatment (17, 18). In addition to several blood transcriptomic signatures (19–23), host plasma proteomic signatures have also been widely evaluated as diagnostic tools for TB, but also for monitoring treatment (24–28).

Various cytokines and chemokines linked to the Th1/Th17 and Th2/Th9 signaling pathways are involved at different stages of the TB spectrum, either to contain infection in the early phase, or to contribute to Mtb elimination in TB disease (29–32). However, a thorough description of the host immune response at different stages of treatment is needed for the development of more sensitive and specific non-sputum-based tests for monitoring TB treatment.

Here, we conducted an observational and cross-sectional cohort study in 32 patients with clinically and microbiologically confirmed PTB, followed up before the start of treatment (T0), after 2 months of treatment (T1) and at the end of treatment (T2). At each timepoint plasma levels of IFN-γ, IL-1717 pro- and anti-inflammatory cytokines/chemokines and complement factors C1q, C3 and C4 were assessed in unstimulated and Mtb-specific stimulated plasma using interferon-γ release assays as QuantiFERON-TB gold *plus* and rmsHBHA.

This study provides new information on host immune responses throughout the course of anti-TB treatment, and enables the identification of a plasma protein signature, composed of C1q and IL-13-specific response to rmsHBHA, capable of differentiating an active form of pulmonary TB from cured disease.

## 2 Materials and methods

### 2.1 Type of study

Cross-sectional and observational clinical evaluation of blood biomarkers for monitoring the efficacy of anti-tuberculosis treatment (HINTT project). The Instituto de Investigaciones en Ciencias de la Salud (Universidad Nacional de Asunción; IICS-UNA) in Asunción, Paraguay, led the project in Paraguay with the support from Mérieux Foundation, Lyon, France.

### 2.2 Ethical considerations

The research obtained ethical approval from the Research Ethics Committee and the Scientific Committee of the IICS-UNA (IRB number: IRB00011984; Federal Wide Assurance number: FWA00029097). Informed written consent was obtained from all enrolled patients. The results obtained did not interfere with the national standards for TB diagnosis and treatment; standard procedures from the National Tuberculosis Program were followed.

### 2.3 Cohort recruitment, TB diagnosis, and patient follow-up

Between July 2018 and June 2020, 32 patients with microbiological confirmed pulmonary TB (positive culture and/or sputum smear and/or GeneXpert MTB/RIF) were enrolled in the study. Among them, 90.6% (29/32) were confirmed by sputum smear-microscopy (Ziehl-Neelsen and/or Auramine O staining). Patients with HIV or HbA1c levels exceeding 6.5%; Cobas (Roche) automated analyzer were excluded. The median age of the patients included in the cohort was 35 years (IQR: 23-49), 56.2% (18/32) of the individuals were male, the median body mass index was 20.5 (IQR: 18.2-22.82), 6 subjects (18.8%) had a history of TB, and 68.8% (22/32) of the subjects had proof of BCG vaccination. At inclusion, all subjects had clinical symptoms consistent with pulmonary TB. Patients were followed up for clinical evaluation before starting anti-TB treatment, at baseline (T0), after 2 months of treatment (T1) and at the end of treatment (T2). All patients in this cohort followed the 6-months WHO-recommended treatment regimen for drug-susceptible TB. Treatment outcomes were defined according to WHO guidelines: cured (negative sputum culture at T2); failed (positive sputum culture at T2). The study flowchart is described in **Supplementary Figure 1**.

### 2.4 Whole blood sampling, cell count and IGRA

At each time point, venous whole blood was collected and processed as follows: 1 ml was collected in EDTA tubes for whole blood cell counting using a standardized automated system, 2 ml were collected in dry tubes for serological analysis, including glutamate-pyruvate transaminase (GPT), γ-glutamyl-transferase (GGT), alkaline phosphatase and HIV testing. 5 ml were collected in lithium heparin tubes for IGRA. QuantiFERON-TB Gold Plus (QFT-P; Qiagen) were performed according to manufacturer’s instructions. RmsHBHA was used in an in-house whole blood stimulation test at a final concentration of 5 µg/ml according previously described method (11, 12, 14, 15). Within 2 hours of blood collection, the samples were placed at 37°C in a 5% CO2 atmosphere and incubated for 24 hours. After incubation, plasma was separated from the cell fraction by decantation and stored at −80°C for biomarker quantification.

### 2.5 Quantification of cytokines and chemokines levels in plasma

Plasma levels of TNFα, IL-2, IL-4, IL-13, IFN-γ, IL-1β, IL-17, IL-6, IL-7, IL-8, IL-12p70, G-CSF, GM-CSF, IL-5, IL-10, MCP-1 and MIP-1β were assessed using the Bio-Plex Pro Human Cytokine 17-Plex (BIO-RAD, USA) in accordance with the manufacturer’s instructions. Plasma C1q/C3/C4 levels were assessed using the HCMP2MAG-19K MILLIPLEX MAP Human Complement Panel 2 assay (Millipore) in accordance with the manufacturer’s instructions. Multiplex readings were performed using MAGPIX^®^ equipment (Luminex platform). In addition, IFN-γ secretion was quantified using the QuantiFERON-TB Gold Plus (QFT-P) ELISA kit (Qiagen), in accordance with the manufacturer’s instructions.

### 2.6 Clinical data collection and statistical analysis

Standardized clinical report formats and data collection forms were used in accordance with the methodology described (33). Data were entered into the CASTOR database system (version 1.4, Netherlands) and then subjected to extensive cleaning and analysis in RStudio (version 1.4.1106). For continuous variables with a non-normal distribution, the Mann-Whitney test was used. In the case of repeated measures involving non-independent continuous variables, analysis was performed using the Friedman rank sum test, followed by the Wilcoxon-Nemenyi-McDonald-Thompson (34) *post-hoc* test. Principal component analysis was performed using the R-studio interface and implemented with the Kassambara script (35). Combinatorial analysis of multiple biomarkers was performed using the CombiROC software package to determine the most effective combinations of the plasma markers studied (36). Combinations with the highest area under the receiver operating characteristic curve (AUC) were considered for the identification of potent immune biomarker signatures. The process involved computational evaluation and selection of the most optimal biomarker combinations via integrated ROC analysis.

## 3 Results

### 3.1 Descriptive analysis of Mtb-specific and non-specific immune responses throughout TB treatment

**Figure 1:**
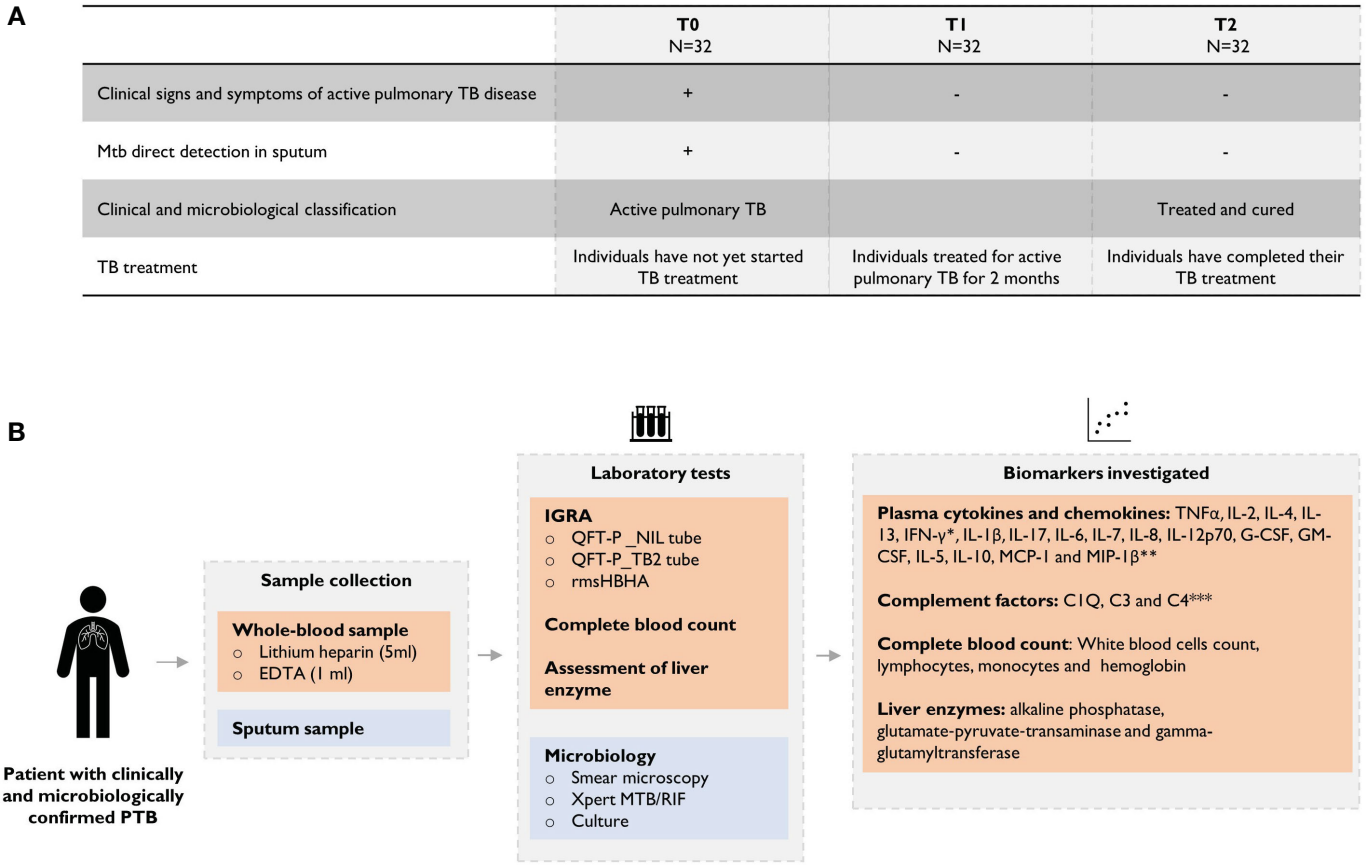
Overview of the study design. **(A)** Clinical and microbiological definition of patients during TB treatment and **(B)** description of analyses performed throughout the study. Plasma levels were assessed using the *QuantiFERON-TB Gold Plus (QFT-P) ELISA kit (Qiagen) or **Bio-Plex Pro Human Cytokine 17-Plex (BIO-RAD, United States) or *** HCMP2MAG-19K MILLIPLEX MAP Human Complement Panel 2 assay (Millipore) on MAGPIX ® equipment (Luminex).

**Figure 2:**
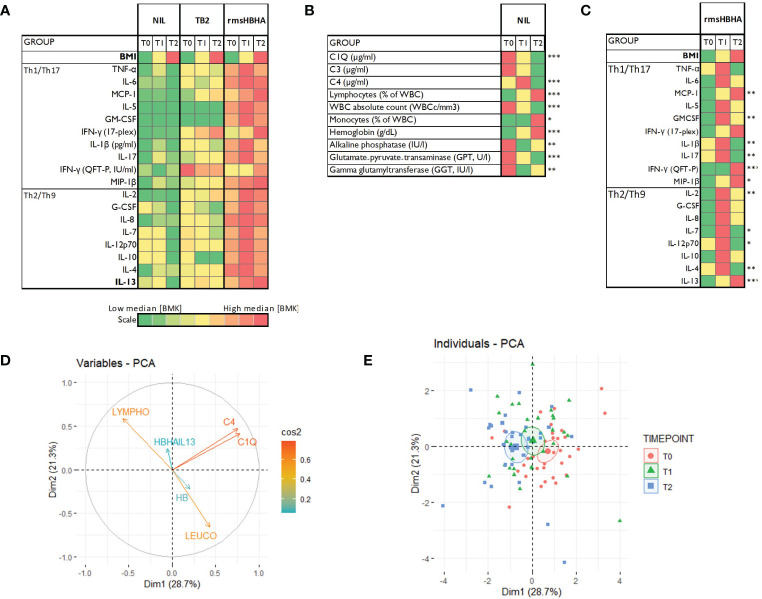
Host blood biomarkers expression during TB treatment and principal component analysis. **(A)**: Heatmaps showing comparison of the median expression of 20 blood biomarkers at baseline (T0), after 2 months of treatment (T1) and at the end of treatment (T2) with unstimulated condition as reference, or in the unstimulated condition only **(B)**, or in the rmsHBHA condition only **(C)**. Principal component analysis (PCA) was performed on the expression of 9 markers. Arrows length represent the contribution of each marker to the variance described by Dim1 and Dim2 **(D)**. PCA representation of different group of patients **(E)**. Each dot represents one patient. Color coding represents the different clinical groups. The axes represent principal components 1 (Dimension 1, Dim1) and 2 (Dimension 2, Dim2) and the percentages indicate their contribution to the total observed variance. Axis values represent individual PCA scores. The concentration ellipses correspond to 100% data coverage. *p < 0.05; **p < 0.01; ***p < 0.001.

The clinical and microbiological classification of patients at each follow-up visit is shown in **Figure 1A**. At each time point, levels of cytokines and chemokines (TNFα, IL-2, IL-4, IL-13, IFN-γ, IL-1β, IL-17, IL-6, IL-7, IL-8, IL-12p70, G-CSF, GM-CSF, IL-5, IL-10, MCP-1, MIP-1β) and complement factors (C1q, C3 and C4) were assessed in unstimulated plasma and Mtb-specific stimulated plasma from blood stimulated by the QuantiFERON-TB gold *plus* TB2 tube or with rmsHBHA (**Figure 1B**). Complete blood count and alkaline phosphatase, glutamate-pyruvate-transaminase (GPT) and γ-glutamyl transferase (GGT) were evaluated in unstimulated whole blood. The expression levels of each plasma or blood biomarker are reported in **Table 1** and summarized in heatmaps in **Figure 2**. Overall, rmsHBHA stimulated blood contained a significant higher concentration of inflammatory mediators compared to unstimulated and TB2 stimulated plasma (**Figure 2A**). C1q (P<0.001) plasma levels, absolute WBC count (P=0.001), alkaline phosphatase (P=0.003), GGT (P<0.001) and GPT (P=0.008) enzymes were significantly higher in unstimulated plasma from patients with PTB at baseline (T0) than other follow-up timepoints (**Figure 2B**). C4 (P<0.001) plasma levels were the highest after 2 months of TB treatment. The proportion of lymphocytes (P<0.001), monocytes (P=0.05) and hemoglobin (P<0.001) were highest at the end of treatment (T2). A macroscopic overview of the immune response to rmsHBHA reveals an overall activation of Th1/Th17 and Th2/Th9 cells signaling pathways after 2 months of TB-treatment (**Figure 2C**).

**Table 1:** Quantification of host blood biomarkers during TB treatment.

		Timepoint			
Condition	Biomarkers	T0	T1	T2	*p*
Unstimulated	MIP-1β	822.5 (447.7 to 2352.7)	1319.6 (931.3 to 3424.8)	1053.5 (580.8 to 2688.8)	*0.150*
	IL-6	64.5 (24.3 to 223.5)	59.0 (13.5 to 230.0)	36.8 (3.6 to 168.1)	*0.519*
	IFN-γ	6.8 (4.2 to 16.6)	8.5 (6.4 to 15.3)	8.3 (6.4 to 17.3)	*0.481*
	IL-5	5.3 (5.3 to 10.9)	5.3 (5.3 to 21.7)	5.3 (5.3 to 6.0)	*0.285*
	GM-CSF	0.4 (0.4 to 0.7)	0.4 (0.4 to 1.2)	0.4 (0.4 to 0.4)	*0.362*
	TNF-α	56.9 (23.6 to 195.6)	84.7 (33.0 to 227.2)	49.4 (24.5 to 105.0)	*0.354*
	IL-2	2.4 (1.7 to 5.6)	3.4 (1.7 to 6.2)	2.7 (1.7 to 8.3)	*0.906*
	IL1-β	10.0 (5.1 to 35.1)	14.9 (7.7 to 60.4)	10.1 (0.8 to 31.1)	*0.440*
	IL-13	0.6 (0.3 to 1.2)	0.7 (0.3 to 1.0)	0.3 (0.3 to 1.1)	*0.473*
	IL-4	2.1 (1.1 to 5.7)	3.0 (1.6 to 6.5)	3.0 (0.4 to 5.0)	*0.618*
	MCP-1	1570.3 (879.2 to 2126.3)	1653.4 (1035.5 to 2474.8)	1629.2 (980.5 to 2306.9)	*0.691*
	IL-8	5197.2 (2386.2 to 18232.2)	7283.6 (3927.7 to 20243.9)	6912.0 (2024.1 to 19465.0)	*0.818*
	IL-10	5.0 (2.3 to 10.7)	5.0 (3.4 to 12.4)	4.8 (0.8 to 6.8)	*0.414*
	G-CSF	54.4 (4.3 to 109.6)	48.5 (7.7 to 80.1)	34.1 (4.3 to 89.2)	*0.807*
	IL-7	0.5 (0.3 to 1.0)	0.5 (0.3 to 1.0)	0.3 (0.3 to 1.0)	*0.644*
	IL-12P70	7.5 (1.9 to 17.1)	7.5 (2.0 to 17.8)	6.3 (1.9 to 20.1)	*0.749*
	IL-17	2.7 (2.7 to 10.9)	6.6 (2.7 to 12.9)	4.3 (2.7 to 9.2)	*0.211*
	C1q	174.1 (149.2 to 196.8)	152.9 (131.3 to 185.9)	131.9 (117.0 to 155.3)	*0.002*
	C3	403.5 (270.3 to 507.0)	369.7 (259.5 to 455.8)	341.5 (232.8 to 473.9)	*0.292*
	C4	584.5 (493.3 to 644.4)	575.2 (470.6 to 623.1)	456.8 (386.1 to 548.1)	*0.005*
TB2	MIP-1β	1367.0 (476.6 to 2228.6)	1563.6 (934.8 to 2887.8)	1894.6 (1029.5 to 2929.3)	*0.164*
	IL-6	105.0 (27.7 to 402.9)	65.9 (23.6 to 564.8)	79.4 (10.2 to 387.5)	*0.928*
	IFN-γ	13.1 (8.7 to 32.8)	14.8 (8.6 to 31.9)	17.0 (12.2 to 36.2)	*0.331*
	IL-5	5.3 (5.3 to 24.1)	5.3 (5.3 to 29.9)	5.5 (5.3 to 17.5)	*0.595*
	GM-CSF	0.4 (0.4 to 1.6)	0.4 (0.4 to 1.8)	0.4 (0.4 to 3.2)	*0.885*
	TNF-α	108.5 (28.0 to 200.9)	93.8 (29.9 to 229.0)	77.0 (39.2 to 169.2)	*0.890*
	IL-2	11.9 (3.7 to 24.6)	11.1 (1.7 to 39.9)	15.8 (6.7 to 45.9)	*0.371*
	IL1-β	18.7 (8.2 to 64.8)	22.2 (4.9 to 49.3)	16.8 (4.2 to 52.7)	*0.984*
	IL-13	0.7 (0.3 to 1.3)	1.0 (0.3 to 1.5)	0.7 (0.3 to 1.8)	*0.567*
	IL-4	3.2 (1.5 to 6.6)	3.3 (1.3 to 6.3)	4.0 (2.2 to 7.6)	*0.930*
	MCP-1	1705.5 (934.5 to 2633.2)	1917.0 (1117.8 to 2894.8)	2202.9 (1688.8 to 2913.9)	*0.329*
	IL-8	10744.7 (3025.0 to 23966.1)	10125.7 (4657.0 to 23719.0)	8875.6 (5343.7 to 31318.7)	*0.812*
	IL-10	6.7 (1.9 to 10.0)	4.6 (2.0 to 8.7)	4.6 (1.6 to 8.4)	*0.865*
	G-CSF	62.3 (4.3 to 112.7)	47.0 (4.3 to 81.8)	33.0 (4.3 to 81.1)	*0.558*
	IL-7	0.6 (0.3 to 1.1)	0.5 (0.3 to 1.0)	0.4 (0.3 to 1.1)	*0.842*
	IL-12P70	10.7 (1.9 to 24.5)	7.5 (1.9 to 25.6)	8.8 (1.9 to 23.1)	*0.871*
	IL-17	5.5 (2.7 to 14.1)	7.2 (2.7 to 13.3)	7.9 (2.7 to 17.7)	*0.489*
rmsHBHA	MIP-1β	6772.6 (3972.5 to 13668.5)	9961.7 (5932.3 to 17003.2)	11643.5 (6857.3 to 17570.3)	*0.105*
	IL-6	4512.4 (2061.3 to 9009.2)	6536.7 (5526.7 to 11812.3)	6805.8 (3062.9 to 10985.8)	*0.082*
	IFN-γ	12.4 (9.1 to 36.2)	14.1 (10.7 to 30.4)	18.0 (12.1 to 32.5)	*0.380*
	IL-5	110.1 (5.3 to 188.1)	186.0 (88.8 to 261.9)	130.2 (15.1 to 262.5)	*0.237*
	GM-CSF	4.2 (0.4 to 13.0)	8.5 (0.6 to 23.3)	6.7 (0.4 to 18.4)	*0.532*
	TNF-α	459.7 (258.6 to 1402.9)	812.8 (449.0 to 2619.9)	498.8 (292.1 to 2119.5)	*0.257*
	IL-2	19.6 (9.0 to 48.8)	37.9 (21.0 to 68.3)	34.6 (8.5 to 67.0)	*0.213*
	IL1-β	420.6 (155.4 to 797.0)	776.7 (406.1 to 4027.9)	285.5 (104.1 to 1140.2)	*0.057*
	IL-13	1.4 (0.5 to 2.2)	2.5 (1.2 to 4.4)	2.9 (0.6 to 4.7)	*0.040*
	IL-4	25.7 (12.3 to 35.5)	29.6 (21.6 to 50.2)	18.5 (11.6 to 43.3)	*0.229*
	MCP-1	2118.1 (1734.2 to 2935.5)	2567.4 (1783.9 to 3597.3)	2865.3 (2409.6 to 3265.2)	*0.055*
	IL-8	44424.2 (31269.5 to 63095.9)	52874.8 (42872.7 to 65119.9)	46264.8 (33589.4 to 51569.8)	*0.099*
	IL-10	60.0 (31.1 to 134.8)	121.4 (58.1 to 214.3)	111.4 (45.6 to 297.8)	*0.119*
	G-CSF	193.9 (115.5 to 519.5)	396.7 (188.0 to 1001.9)	288.6 (117.2 to 1011.1)	*0.292*
	IL-7	1.0 (0.5 to 2.0)	1.6 (0.4 to 2.6)	1.5 (0.3 to 2.9)	*0.436*
	IL-12P70	27.4 (11.6 to 42.3)	42.7 (9.6 to 64.8)	30.0 (1.9 to 56.8)	*0.448*
	IL-17	56.8 (25.0 to 102.9)	91.1 (52.3 to 191.7)	40.2 (20.0 to 130.4)	*0.086*
Unstimulated	WBCc	9665.0 (7582.5 to 11640.0)	7570.0 (6150.0 to 9260.0)	6375.0 (5252.5 to 8205.0)	0.001
	Lymphocytes	18.0 (14.8 to 23.0)	24.0 (19.0 to 31.0)	29.5 (23.5 to 37.0)	<0.001
	Monocytes	0.5 (0.0 to 2.0)	0.0 (0.0 to 2.0)	2.0 (0.0 to 2.0)	0.332
	Hemoglobin	11.8 (10.6 to 13.1)	13.3 (12.5 to 14.1)	13.4 (12.7 to 15.1)	<0.001
	PA	137.5 (77.5 to 169.0)	100.0 (91.0 to 125.0)	102.0 (74.5 to 114.8)	0.299
	GPT	20.0 (12.5 to 31.0)	12.0 (10.0 to 20.0)	9.0 (8.0 to 11.0)	0.001
	GGT	74.5 (34.2 to 90.0)	35.0 (28.0 to 72.0)	32.0 (21.0 to 54.5)	0.083

T0, baseline; T1, 2 months of treatment; T2, end of treatment. Condition refers to the IGRA, unstimulated, TB2 from the QuantiFERON-TB gold plus assay and rmsHBHA for the in-house IGRA. PA, alkaline phosphatase; GPT, glutamate‐pyruvate‐transaminase; GGT, γ -glutamyl transferase.

### 3.2 Identification of the main parameters discriminating active pulmonary TB (T0) from a resolved infection (T2)

Plasma cytokines/chemokines, complement factors, blood counts and levels of liver enzymes were included in a principal component analysis (PCA) to identify the main parameters associated with the specific phases of TB treatment (T0, T1 and T2). PCA results showed that complement factors C1q and C4 plasma levels, lymphocyte proportion, absolute WBC count, body mass index (BMI) and hemoglobin (HB) were the main variables contributing most to differentiating a PTB at onset from a cured TB profile (**Figures 2D, E**).

**Figure 3:**
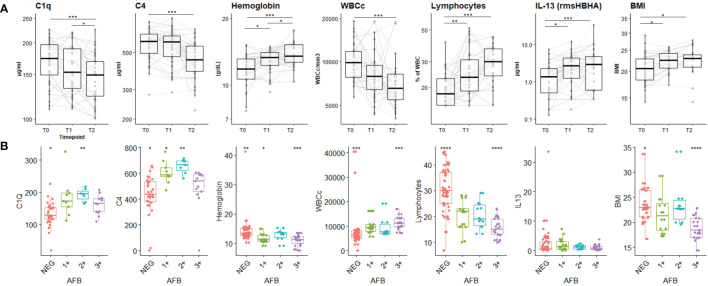
Plasma concentrations of the main biomarkers of interest during the TB-treatment period and stratified according to sputum Mtb load. **(A)** Expression dynamics of the PCA-selected biomarkers during TB treatment. T0: before treatment; T1: after two months of treatment; T2: at the end of treatment. The boxplot shows the median (bold bar) and interquartile range (thin rectangle). Lines show data for each patient. WBCc: absolute white blood cell count. BMI: body mass index. Repeated measures of non-independent continuous variables were analyzed using the Friedman rank-sum test, with Wilcoxon-Nemenyi-McDonald-Thompson’s *post-hoc* test. **(B)** Plasma concentration of PCA-selected biomarkers according to sputum Mtb load. AFB: acid-fast bacilli detected by sputum smear microscopy. 1+: AFB smear grade 1+; 2+: AFB smear grade 2+; 3+: AFB smear grade 3+; NEG: negative detection by AFB smear microscopy. Statistical significance was calculated using Mann–Whitney U test. *p < 0.05; **p < 0.01; ***p < 0.001.

The dynamic expression of the main biomarkers of interest identified in the PCA are shown in **Figure 3**. Levels of C1q, C4, absolute WBC count decreased significantly over time, while hemoglobin levels, lymphocyte proportion, IL-13 response to HBHA (IL-13rmsHBHA) and BMI increased significantly over the same period (**Figure 3A**). Plasma C1q and C4q levels were significantly lower in PTB individuals with a negative sputum smear microscopy than in those with positive (grade 1+ and 2+) sputum smear microscopy (**Figure 3B**).

### 3.3 Identification of a host blood signature to differentiate PTB at diagnosis or during disease from cured profiles

CombiROC algorithm was first used to identify the best combinations of plasma host markers to distinguish PTB profiles (T0, sputum culture positive at baseline) from cured profiles (T2, sputum culture negative at the end of treatment) (**Table 2**). A set of 57 signatures were obtained from the six selected markers. These signatures were ranked according to their decreasing AUC values, then, the number and the relevance of the combined markers involved in each signature. The highest AUC for a single marker was obtained with lymphocytes proportion as biomarker for differentiating PTB disease at diagnosis from cured profiles. A signature including levels of C1q and hemoglobin, combined with an evaluation of the levels of IL-13 in response to rmsHBHA concentration can discriminate PTB disease at diagnosis from cured profiles with an AUC of 0.92, sensitivity of 94%, specificity of 79%. A second analysis aimed to identify and evaluate the analytical performance of a protein signature capable of differentiating subjects according to their bacterial load (**Supplementary Table 1**). The [C1q] + [HB] + [IL-13rmsHBHA] signature showed an AUC of 0.84, sensitivity of 89% and specificity of 78% in distinguishing subjects with a low bacterial load (grade 1+) from microscopy-negative subjects.

**Table 2:** Accuracy of single or biomarker combinations for differentiating PTB disease at diagnosis (T0) from treated and cured profiles (T2).

Biomarkers	AUC	SE	SP	CutOff	ACC	NPV	PPV
Body Mass Index (BMI)	0.65	60%	67%	0.53	63%	60%	67%
Hemoglobin (HB; g/dL)	0.79	53%	93%	0.68	72%	63%	90%
WBC absolute count (WBC_c_;/mm3)	0.77	63%	86%	0.54	73%	67%	83%
Lymphocytes (% of WBC)	0.81	81%	75%	0.50	78%	78%	79%
[C1q]_unstimulated condition_	0.77	69%	79%	0.60	73%	69%	79%
[C4]_unstimulated condition_	0.74	59%	86%	0.55	72%	65%	83%
[IL-13]_rmsHBHA_	0.66	78%	61%	0.51	70%	71%	69%
[C1q] + [HB] + Lymphocytes + [IL-13]_rmsHBHA_	0.93	78%	96%	0.67	87%	79%	96%
[C1q] + [C4]+ [HB] + [IL-13]_rmsHBHA_	0.93	88%	89%	0.60	88%	86%	90%
[C1q] + [HB] + [IL-13]_rmsHBHA_	0.92	94%	79%	0.41	87%	92%	83%

AUC, Area Under the Receiver Operating Characteristic Curve (AUC; ROC); SE, sensitivity; SP, specificity; ACC, accuracy; NPV, negative predictive value; PPV, positive predictive value.

## 4 Discussion

In the present study we characterized the immune responses of patients with microbiologically confirmed PTB at different timepoints of the treatment regimen to identify host blood signatures that distinguish individuals with PTB disease at enrolment from those with cured disease. We demonstrated that host C1q plasma levels and IL-13-specific response to rmsHBHA differentiate active form of pulmonary TB from cured disease.

Levels of plasma complement factors C1q and C4 were significantly higher at baseline, in patients with clinically and microbiologically confirmed PTB than in subjects fully treated and cured. These results suggest that complement factors are highly involved during the active phase of the TB and less during treatment phase according to the significant decrease of those two biomarkers across TB-treatment. These findings align with existing literature, wherein the C1q protein has been newly identified as a diagnostic biomarker for tuberculous pleural effusion (TPE), demonstrating notably high diagnostic accuracy, particularly among younger patients (37). In non-human primates (NHP), elevated levels of C1q, whether observed systemically or within the local environment, are positively correlated with the advancement of TB disease (assessed by PET-CT imaging or post-mortem evaluation (38). In a separate cohort of South African adolescents with latent Mtb infection, a specific gene pair, C1qC/TRAV27, has been identified as a consistent predictor of TB progression (39). Notably, this prediction holds true for household contacts across various African sites yet does not apply to infected adolescents without identifiable recent exposure events. In a different study, elevated levels of C1q were observed in individuals with TB disease when compared to those with latent TB infection, as well as patients with sarcoidosis, leprosy, and pneumonia (40). A deficiency in complement C4 could potentially pose as a risk factor for non-tuberculous mycobacteria (NTM) infection among individuals appearing to be immunocompetent (41). In addition, C1q and C4 were differentially expressed in plasma of PTB patients according to Mtb bacterial load assessed by smear microscopy at baseline. This later finding implies that assessing the quantitative levels of C1q in unstimulated plasma could hold promise in enhancing the diagnosis of paucibacillary forms of TB (such as childhood TB and/or extra-pulmonary TB).

Immune specific responses using rmsHBHA induced a higher production of inflammatory and anti-inflammatory mediators compared to the Mtb-specific peptides included in TB2 tube from QFT. The highest accuracy for TB stage discrimination was associated with IL-13 response to rmsHBHA which significantly increased in cured TB suggesting that cure is associated with an anti-inflammatory status.

Moreover, we observed a time-dependent engagement of Th1/Th17 and Th2/Th9 cell signaling pathways and distinct cytokine and chemokine profiles at three different timepoints throughout the treatment regimen.

Host plasma C1q and the detection of IL-13 levels in response to rmsHBHA levels in addition to hemoglobin evaluation in whole blood allowed to discriminate patients with PTB (T0) from those who have resolved the disease (T2) with an AUC of 0.92, sensitivity of 94%, specificity of 79%. These results, although preliminary, would meet the accuracy expectations for a treatment monitoring assay, as recently defined in the WHO’s Target Product Profile (TPP) (42). This host blood signature could be evaluated as part of clinical trials aimed at shortening anti-TB treatments, or as part of monitoring MDR-TB patients throughout the course of their treatment.

As C1q levels correlates with Mtb load, we suggest this blood assay could be of interest for detecting paucibacillary and/or subclinical forms of TB. This approach could enhance TB prevention by identifying the beneficiaries of the TB preventive treatment among high-risk group populations.

The logistics beyond laboratory testing using IGRA approach remains a major limitation (requirement of blood sample collection, transfer to the laboratory, technical processing, and analysis), which to date prevents it from being deployed in the field, for use in decentralized diagnostic approaches or in household contact surveys or active case finding campaigns.

This study has few limitations. The evaluation of the immune response to rmsHBHA and TB2 across TB-treatment was performed only in one of the four sites of the HINTT multicentered project. The different signatures and their accuracy found in this nested study in Paraguay, from a relatively small sample size (32 subjects), need to be validated in different and larger cohorts. Moreover, we could not evaluate the ability of this signature to predict treatment failure because all patients were successful treated. Finally, we included only drug-susceptible TB because no MDR-TB cases were enrolled.

In conclusion, our results suggest the combined assays based on the quantitative evaluation of IL-13 in response to rmsHBHA and plasma C1q levels are a promising tool for TB treatment monitoring. These host markers may be valuable in the initial identification of patients who would benefit from vigilant monitoring during e treatment.

## Data availability statement

The raw data supporting the conclusions of this article will be made available by the authors, without undue reservation.

## Ethics statement

The studies involving humans were approved by Research Ethics Committee and the Scientific Committee of the IICS-UNA (IRB number: IRB00011984; Federal Wide Assurance number: FWA00029097). The studies were conducted in accordance with the local legislation and institutional requirements. The participants provided their written informed consent to participate in this study.

## Author contributions

GR: Conceptualization, Funding acquisition, Methodology, Project administration, Writing – review & editing, Resources. DS: Writing – review & editing, Data curation, Formal analysis, Investigation. CCDA: Data curation, Formal analysis, Investigation, Writing – review & editing. LR: Investigation, Writing – review & editing. LF: Investigation, Writing – review & editing. RA: Investigation, Writing – review & editing. GD: Conceptualization, Resources, Writing – review & editing. MDBN: Formal analysis, Writing – review & editing. RB: Formal analysis, Writing – review & editing. NR: Formal analysis, Writing – review & editing. DG: Conceptualization, Funding acquisition, Methodology, Writing – review & editing. JH: Conceptualization, Funding acquisition, Methodology, Project administration, Writing – original draft, Writing – review & editing.
