# Supplementary figures and images for: C1q and HBHA-specific IL-13 levels as surrogate plasma biomarkers for monitoring tuberculosis treatment efficacy: a cross-sectional cohort study in Paraguay
Source: Front Immunol. 2024 Mar 13;15:1308015. doi: 10.3389/fimmu.2024.1308015 (PMC10967656; doi:10.3389/fimmu.2024.1308015)

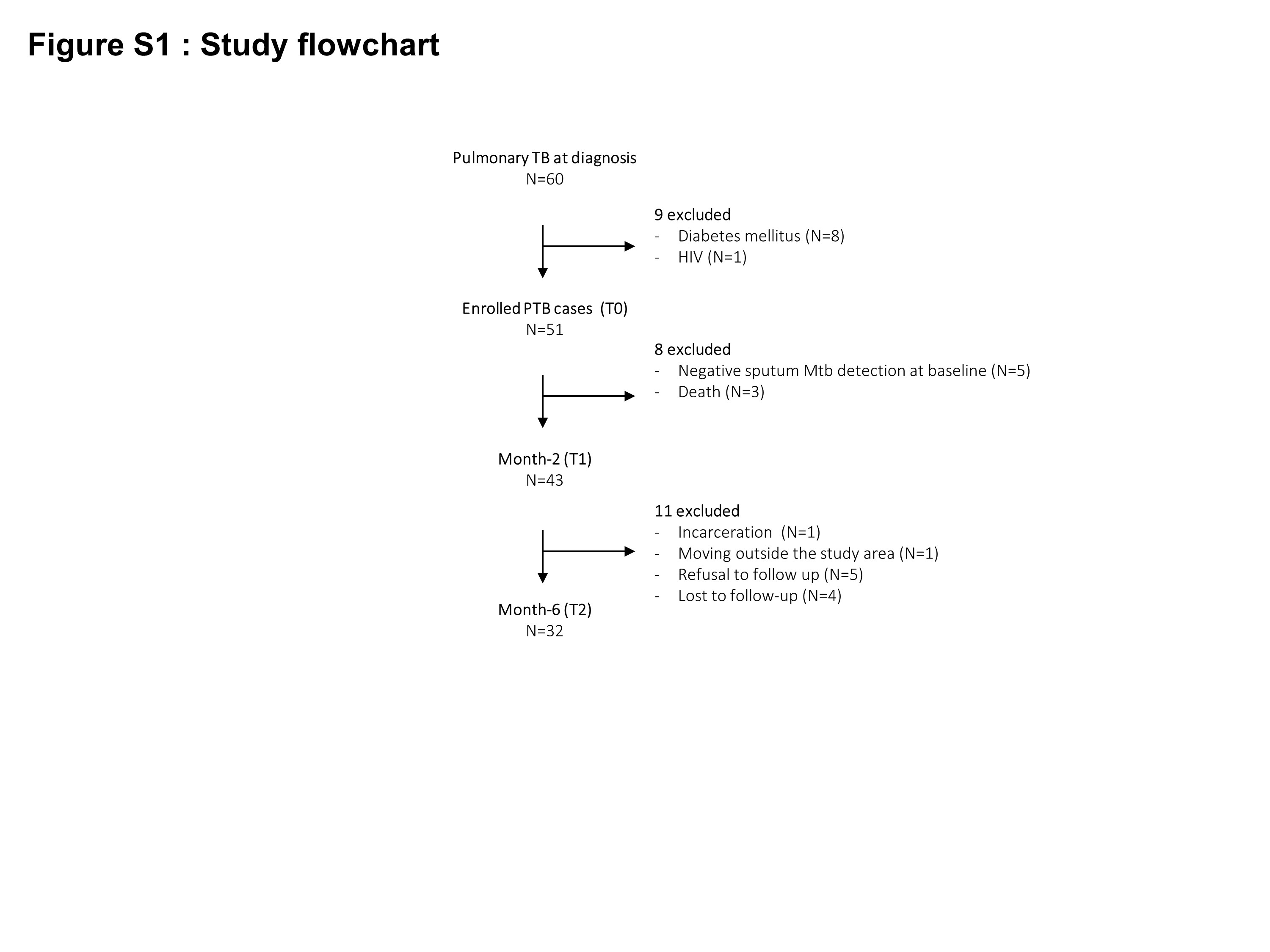

Supplement: Supplementary file 1 [file Image_1.jpeg]
