# Supplementary material for: C1q and HBHA-specific IL-13 levels as surrogate plasma biomarkers for monitoring tuberculosis treatment efficacy: a cross-sectional cohort study in Paraguay
Source: Front Immunol. 2024 Mar 13;15:1308015. doi: 10.3389/fimmu.2024.1308015 (PMC10967656; doi:10.3389/fimmu.2024.1308015)
Supplement: Supplementary file 2 [file Table_1.docx]

**Table S1: Accuracy of single or biomarker combinations for differentiating subjects with various Mtb load (assessed by smear-microscopy).**

| Biomarkers | AUC | SE | SP | CutOff | ACC | NPV | PPV |
| --- | --- | --- | --- | --- | --- | --- | --- |
|  |  |  |  |  |  |  |  |
| *Negative vs Low-grade (1+) positive SM* |  |  |  |  |  |  |  |
| [C1q] | 0.72 | 78% | 67% | 0.12 | 68% | 95% | 26% |
| [C4] | 0.74 | 78% | 68% | 0.16 | 70% | 95% | 27% |
| Hemoglobin (HB; g/dL) | 0.78 | 67% | 90% | 0.23 | 87% | 95% | 50% |
| WBC absolute count (WBC_c_; /mm3) | 0.75 | 67% | 80% | 0.14 | 78% | 94% | 33% |
| Lymphocytes (% of WBC) | 0.75 | 100% | 43% | 0.09 | 51% | 100% | 21% |
| IL-13 | 0.49 | 56% | 60% | 0.13 | 59% | 90% | 17% |
|  |  |  |  |  |  |  |  |
| [C4] + [HB]+ WBCc + [IL-13]rmsHBHA | 0.90 | 78% | 90% | 0.30 | 88% | 96% | 54% |
| [C4] + [HB]+ Lymphocytes + [IL-13]rmsHBHA | 0.90 | 89% | 83% | 0.20 | 84% | 98% | 44% |
| [C4] + [HB]+ Lymphocytes | 0.89 | 100% | 73% | 0.10 | 77% | 100% | 36% |
| [C1q] + [C4] + [HB] + Lymphocytes |  |  |  |  |  |  |  |
| [C1q] + [HB] + [IL-13]_rmsHBHA_ | 0.84 | 89% | 78% | 0.13 | 80% | 98% | 38% |
|  |  |  |  |  |  |  |  |
| *Negative vs High-grade (2+/3+) positive SM* |  |  |  |  |  |  |  |
| [C1q] | 0.71 | 75% | 68% | 0.27 | 70% | 89% | 44% |
| [C4] | 0.65 | 65% | 65% | 0.25 | 65% | 85% | 38% |
| Hemoglobin (HB; g/dL) | 0.74 | 60% | 82% | 0.27 | 76% | 86% | 52% |
| WBC absolute count (WBC_c_; /mm3) | 0.77 | 100% | 47% | 0.19 | 60% | 100% | 39% |
| Lymphocytes (% of WBC) | 0.83 | 90% | 65% | 0.19 | 71% | 95% | 46% |
| IL-13 | 0.69 | 95% | 52% | 0.20 | 63% | 97% | 40% |
|  |  |  |  |  |  |  |  |
| [C1q] + [HB] + WBCc + [IL-13]_rmsHBHA_ | 0.93 | 100% | 77% | 0.15 | 83% | 100% | 59% |
| [C1q ]+ [HB] + Lymphocytes + [IL-13]rmsHBHA | 0.93 | 100% | 75% | 0.16 | 81% | 100% | 57% |
| [C1q] + [C4] + [HB] + [IL-13]rmsHBHA | 0.92 | 85% | 88% | 0.30 | 88% | 95% | 71% |
| [C1q] + [HB] + [IL-13]_rmsHBHA_ | 0.91 | 100% | 68% | 0.14 | 76% | 100% | 51% |
|  |  |  |  |  |  |  |  |
| *Low-grade (1+) vs High-grade (2+/3+) positive SM* |  |  |  |  |  |  |  |
| [C1q] | 0.49 | 22% | 90% | 0.27 | 69% | 72% | 50% |
| [C4] | 0.59 | 78% | 45% | 0.31 | 55% | 82% | 39% |
| Hemoglobin (HB; g/dL) | 0.55 | 67% | 60% | 0.31 | 62% | 80% | 43% |
| WBC absolute count (WBC_c_; /mm3) | 0.53 | 78% | 45% | 0.30 | 55% | 82% | 39% |
| Lymphocytes (% of WBC) | 0.64 | 56% | 80% | 0.38 | 72% | 80% | 56% |
| IL-13 | 0.68 | 56% | 95% | 0.44 | 83% | 83% | 83% |
|  |  |  |  |  |  |  |  |
| [C4] + [HB]+ Lymphocytes + [IL-13]rmsHBHA | 0.77 | 89% | 55% | 0.21 | 66% | 92% | 47% |
| [HB]+ Lymphocytes + [IL-13]rmsHBHA | 0.77 | 78% | 70% | 0.27 | 72% | 88% | 54% |
| [C1q] +Lymphocytes + [IL-13]rmsHBHA | 0.76 | 56% | 90% | 0.44 | 79% | 82% | 71% |
| [C1q] + [HB] + [IL-13]_rmsHBHA_ | 0.67 | 56% | 95% | 0.46 | 83% | 83% | 83% |

SM: smear microscopy: AUC: Area Under the Receiver Operating Characteristic Curve (AUC; ROC); SE: sensitivity; SP: specificity; ACC: accuracy; NPV: negative predictive value; PPV: positive predictive value.
